# Design and Synthesis of New Benzimidazole and Pyrimidine Derivatives as *α*-glucosidase Inhibitor

**Published:** 2015

**Authors:** Akbar Mobinikhaledi, Behvar Asghari, Mahsa Jabbarpour

**Affiliations:** a *Department of Chemistry, Faculty of Science, Arak University, Arak, Iran.*; b *Department of Plant Production and Breeding Faculty of Engineering and Technology, Imam Khomeini International University, Qazvin, Iran.*

**Keywords:** Benzimidazole, Pyrimidine, Amino acids, *α*-glucosidase inhibition, Antihyperglycemic activity

## Abstract

In an endeavor to find a novel series of antihyperglycemic agents, new benzimidazole and pyrimidine derivatives were successfully synthesized efficiently in high yield with high purity, starting from amino acids in the presence of phosphorus oxychloride (POCl_3_). The synthesized compounds were identified by ^1^H-NMR, ^13^C-NMR, FT-IR spectroscopic techniques and elemental analysis. All products were assayed for their inhibitory effects on yeast and rat intestinal *α*-glucosidases. The results revealed that compounds with aromatic amino acids moiety showed significant inhibition activity on the tested enzymes. Among the benzimidazole derivatives 4c and 4d exhibited the best activity against both of the tested enzymes. Also, among the pyrimidine derivatives 5c and 5d possessed significant inhibition action on the enzymes. The IC_50_ values for the most potent benzimidazole yeast and intestinal *α*-glucosidases inhibitor (4d) were found to be 9.1 and 36.7 µM, respectively. The IC_50_ values for the inhibition of yeast and intestinal *α*-glucosidases by the most active pyrimidine compound (5d) were calculated to be 8.3 and 21.8 µM, respectively. Overall, this study proved that benzimidazole and pyrimidine derivatives with aromatic amino acids moieties can represent novel promising *α*-glucosidase inhibitors.

## Introduction

Diabetes mellitus is the most common metabolic disorder worldwide and is becoming the third leading cause of death, after cancer and cardiovascular diseases (1). According to the published research results the total number of people with diabetes is projected to rise from 171 million in 2000 to 366 million in 2030 (2). Two types of diabetes are currently known: type 1 is insulin-dependent diabetes and type 2 is non-insulin-dependent diabetes (3). In type 1 diabetes the insulin-producing beta cells of the pancreas are destroyed. This type of diabetes can appear at any age and is trigged by environmental factors such as viruses, diet or chemicals in people genetically predisposed. People with type 1 diabetes must inject themselves with insulin and follow a careful diet and exercise (4). Type 2 diabetes is the most common form of diabetes, affecting 85-90% of all diabetic population. This type of diabetes is strongly genetic in origin but life style factors such as excess weight, inactivity, high blood pressure and poor diet are major risk factors for its development. It has been demonstrated that maintenance of healthy blood glucose level is very important for treating type 2 diabetes (5). Several categories of drugs, including sulfonylureas, biguanids, thiazolidinediones, meglitinides and *α*-glucosidase inhibitors are used for type 2 diabetes (6). *α*-Glucosidase inhibitors decrease the postprandial hyperglycemia by inhibition of carbohydrate hydrolyzing enzymes such as *α*-glucosidase and *α*-amylase (7). Acarbose, miglitol and voglibose are the therapeutic drugs that are used as *α*-glucosidase inhibitors in current medical, but due to their deleterious side effects, such as abdominal distention, bloating and diarrhea, the efforts for finding and synthesis of new effective and safer inhibitors has continued (8,9). 

Over the past years, several benzimidazole and pyrimidine derivatives have been synthesized and widely screened for their biological activities. These classes of hetrocycles have found applications in diverse pharmacological areas such as antibacterial, antibiotics (10,11), anticancer (12), anti-inflammatory (13), antifungal (14), antidiabetic (15), enzyme inhibitors (16,17), anti-hepatitis C (18), cytotoxicity (19) and antihypertensive (20). More over these derivatives were applied as antiparasitic, elastase inhibitors, anti-stress, antioxidant, antiviral (anti-HIV), anticonvulsant and antiprotozoal (21-29). Because of this wide range of biological and pharmaceutical activities and industrial applications, benzimidazole and pyrimidine moieties have received much attention in developing new therapeutic agents. Several methods are available for the benzimidazole synthesis. The more attractive method of benzimidazoles synthesis involves cyclocondensation of an aldehyde with substituted 1,2-phenylenediamines in the presence of an acidic catalyst as dehydrating agent. Pyrimidine derivatives were synthesized by different procedures such as biginelli reaction and the other multi component reactions (30-38). In addition this structure was synthesized by the reaction of 1,8-naphtalendiamine with an aldehyde in the presence of an acidic catalyst (39). 

In the light of above observations, we have recently performed some study including the synthesis of some benzimidazole and pyrimidine derivatives (40,41). Results of these investigations have persuaded us to apply some structural modifications to progress their biological activities. So, we have designed some synthesis strategies that include the synthesis of benzimidazole and pyrimidine systems containing protected amino acids. To date there are no reports available describing synthesis and *α*-glucosidase inhibition activities of these classes of heterocycles. Phosphorus oxychloride was necessary for this condensation, which activate the carboxyl group of amino acids and increases its electrophilicity to enhance the addition of diamine.

## Experimental

All reagents and solvents used were purchased from commercial suppliers (Merck, Across, Fluka or Sigma-Aldrich) without further purification. Melting points (M.p.) were taken by using an Electrothermal WRS-1B apparatus. Infrared (FT-IR) spectra were recorded on a Galaxy Series FT-IR spectrometer by using KBr pellets. The ^l^H and ^13^C-NMR spectra were determined in DMSO-*d*_6_ as solvent on an Avance III spectrometer operating at 500 MHz (Bruker Biospin, Fällanden, Switzerland) in which chemical shifts (ppm) were referenced to the internal standard tetramethylsilane (TMS). Elemental analyses were determined on Elemental Analyzer (Elemental, Vario EL III) at the Arak University. Absornance of enzyme assay reaction mixture was recorded by Biotek microplate reader (XS2).

*Synthesis of the protected amino acid (1a-e)*

Into a 100 mL round bottom flask, 10 mmol of amino acid (1a-e), 10 mmol of phthalic anhydride (2) and 20 mL glacial acetic acid were added. If glacial acetic acid did not solve the compounds completely, pyridine would add drop by drop until the mixture substances were solved totally. The mixture was stirred at room temperature overnight and then refluxed for 4 h at 90-100 ^°^C. Finally the solvent was removed under reduced pressure, then was cooled and poured onto crushed ice. The precipitate was filtered off and washed with cold water to eliminate the extra amino acid. The separated compound was recrystallized from ethanol to afford protected amino acid (3a-e):

**Scheme 1:**
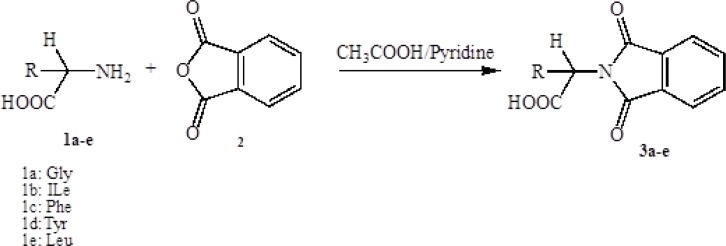
Synthesis of N-protected amino acids.

**Table 1:** Synthesis of N-phthaloyl amino acid

**product**	**Yield (%)**	**Melting point (°C)** **Found reported**		**Colour**
3a	98	199-201	197-198(42)	white
3b	85	127-129	----	white
3c	96	181-183	180-182	milky
3d	97	164-166	162-164	cremy
3e	90	115-117	----	white

*2-(1,3-dioxoisoindolin-2-yl)acetic acid* *(3a)*

IR (KBr, cm^–1^): ν_max_ 3563, 3092, 2992, 2936, 1773, 1726, 1609, 1418, 1319, 1219. 

*3-methyl-2-(1,3-dioxoisoindolin-2-yl)pentanoic acid* *(3b)*

IR (KBr, cm^–1^): ν_max_ 3420, 3120, 2949, 2880, 1775, 1736, 1648, 1485, 1389, 1262.

*2-(1,3-dioxoisoindolin-2-yl)-3-phenylpropanoic acid* *(3c)*

IR (KBr, cm^–1^): ν_max_ 3424, 3067, 3030, 2949, 1773, 1715, 1638, 1487, 1389, 1260.

*3-(4-hydroxyphenyl)-2-(1,3-dioxoisoindolin-2-yl)propanoic acid* *(3d) *

IR (KBr, cm^–1^): ν_max_ 3374, 3067, 1775, 1730, 1611, 1515, 1381, 1227.

*4-methyl-2-(1,3-dioxoisoindolin-2-yl)pentanoic acid* *(3e)*

IR (KBr, cm^–1^): ν_max_ 3482, 3230, 2938, 2884, 1778, 1746, 1694, 1466, 1389, 1283.

*General procedure of preparation of* *2-substituted benzimidazole*

1 mmol* O-*phenydiamine, 1 mmol protected amino acids (3a-d) and 5 mL POCl_3_ were added to 50 mL bottom flask. Then the mixture was refluxed for 4 hours. After completion the reaction cooled at room temperature and poured onto crushed ice. Then brought the alkaline to pH 6-8 with aqueous Potassium carbonate solution. Separated product was collected by filtration and washed with hot ethanol and benzene. Melting points of all products were >300 ^°^C.

**Scheme 2:**
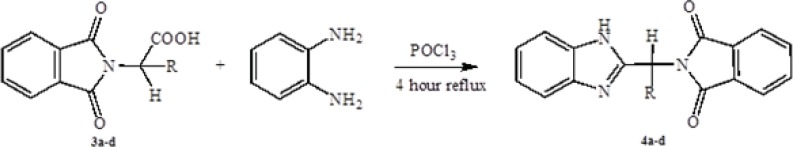
Synthesis of 2-substituded benzimidazole.

*2-((1H-benzo(d)imidazol-2-yl)methyl)isoindoline-1,3-dione* *(4a)*

Brown solid; Yield 96%; IR (KBr): ν_max_ 3379, 3235, 3092, 2918, 1776, 1722, 1628, 1393, 1221. ^1^H-NMR (DMSO-*d*_6_, ppm): δ 12.50 (s, 1H, N-H), 7.95-7.16 (m, 8H, H_aromatic_), 5.03 (s, 2H, CH_2_).^ 13^C-NMR (DMSO-*d*_6_): 168.0, 150.1, 135.2, 132.3, 131.3, 129.1, 123.8, 122.5, 36.0. Anal. calc. for: C_16_H_11_N_3_O_2_, C, 69.31; H, 4.00; N, 15.15 %. Found: C, 69.01; H, 3.79; N, 15.4%.

*2-(1-(1H-benzo(d)imidazol-2-yl)-2-methylbutyl)isoindoline-1,3-dione* *(4b)*

Dark green solid; Yield 84%; IR (KBr): ν_max_ 3433, 3237, 3094, 2970, 2890, 1767, 1716, 1628, 1385, 1221. ^1^H-NMR (DMSO-*d*_6_, ppm): δ 12.46 (s, 1H, N-H), 7.85-7.10 (m, 8H, H_aromatic_), 5.67-5.50 (m, 1H, CH-N), 1.22-0.89 (m, 9H, CH, CH_2_, CH_3_)^ 13^C-NMR (DMSO-*d*_6_, ppm): 167.0, 134.8, 134.2, 131.1, 129.0, 128.5, 128.4, 123.4, 53.5, 53.4, 51.6, 34.2, 34.1. Anal. calc. for: C_20_H_19_N_3_O_2_ C, 72.05; H, 5.74; N, 12.60 %. Found: C, 71.88; H, 5.95; N, 12.76 %.

*2-(1-(1H-benzo(d)imidazol-2-yl)-2-phenylethyl)isoindoline-1,3-dione* *(4c)*

Milky solid; Yield 98%; IR (KBr): ν_max_ 3368, 3061, 3030, 2918, 1776, 1713, 1605, 1429, 1383, 1273. ^1^H-NMR (DMSO-*d*_6_, ppm): δ 12.57 (s, 1H, N-H), 7.84-7.12 (m, 13H, H_aromatic_), 5.86-5.82 (dd, 1H, CH-N), 3.96-3.95 (d, 1H, CH_2_), 3.93-3.92 (d, 1H, CH_2_),^ 13^C-NMR (DMSO-*d*_6_, ppm): 168.4, 139.0, 137.5, 135.8, 134.7, 131.0, 128.8, 128.3, 123.2, 122.3, 112.8, 111.3, 49.9, 35.1. Anal. calc. for: C_23_H_17_N_3_O_2_ C, 75.19; H, 4.66; N, 11.44 %. Found: C, 75.45; H, 4.86; N, 11.73.

*2-(1-(1H-benzo(d)imidazol-2-yl)-2-(4-hydroxyphenyl)ethyl)isoindoline-1,3-dione* *(4d)*

Khaki solid; Yield 82%; IR (KBr): ν_max_ 3383, 3254, 3069, 2934, 1774, 1713, 1611, 1389, 1209. ^1^H-NMR (DMSO-*d*_6_, ppm): δ 12.01 (s, 1H, N-H) 10.91 (s, 1H, O-H), 7.83-6.53 (m, 12H, H_aromatic_), 5.81-5.71 (m, 1H, CH-N), 5.10-4.97 (m, 2H, CH_2_).^ 13^C-NMR (DMSO-*d*_6_, ppm): 167.9, 156.3, 152.5, 135.3, 135.1, 131.2, 131.6, 130.2, 127.7, 123.8, 122.2, 120.2, 118.8, 115.6, 53.9, 33.7. Anal. calc. for: C_23_H_17_N_3_O_3_ C, 72.05; H, 4.47; N, 10.96 %. Found: C, 71.82; H, 4.66; N, 11.20 %.

*General procedure of preparation of 2-substituted pyrimidine*

To a mixture of 1 mmol 1,8-naphtalendiamine and 1 mmol N-phthaloyl-L-amino acids were added 5 mL POCl_3_ in 50 mL bottom flask. Then the mixture was refluxed for 4 hours. After completion the reaction, cooled at room temperature and poured onto crushed ice. Then brought the alkaline to pH 6-8 with aqueous Potassium carbonate solution. Separated product was filtered and washed with hot ethanol and benzene.

**Scheme 3:**
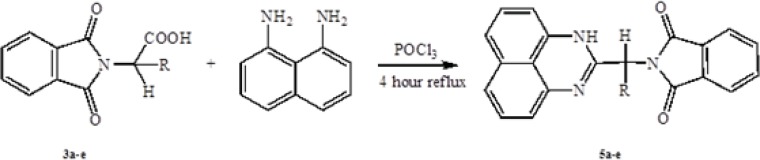
Synthesis of 2-substituted pyrimidine

*2-((1H-perimidin-2-yl)methyl)isoindoline-1,3-dione* *(5a)*

Brown solid, Yield 98 %; IR (KBr): ν_max_ 3376, 3248, 3080, 1719, 1665, 1624, 1557, 1420, 1242. ^1^H-NMR (DMSO-*d*_6_, ppm): δ 8.56 (s, 1H, N-H), 7.96-6.49 (m, 10H, H_aromatic_), 4.54 (s, 2H, CH_2_).^ 13^C-NMR (DMSO-*d*_6_, ppm): 167.8, 135.1, 134.9, 132.4, 129.0, 123.9, 121.9, 120.8, 108.6, 38.2. Anal. calc. for: C_20_H_13_N_3_O_2_ C, 73.38; H, 4.00; N, 12.84 %. Found: C, 73.15; H, 4.18; N, 12.70 %.

*2-(2-methyl-1-(1H-perimidin-2-yl)butyl)isoindoline-1,3-dione* *(5b)*

Brown solid, Yield 98%; IR (KBr): ν_max_ 3387, 3219, 2965, 2876, 1711, 1593, 1360, 1178. ^1^H-NMR (DMSO-*d*_6_, ppm): δ 8.26 (s, 1H, N-H), 7.90-6.09 (s, 10H, H_aromatic_), 5.30-5.27 (m, 1H, CH-N), 2.47-2.45 (m, 1H, CH-CH_3_), 1.30-1.29 (m, 2H, CH_2_-CH_3_), 1.18 (m, 3H, CH-CH_3_), 1.02-0.99 (m, 3H, CH_2_-CH_3_).^ 13^C-NMR (DMSO-*d*_6_, ppm): 167.8, 153.3, 134.8, 134.7, 134.6, 131.4, 131.2, 129.8, 123.3, 123.1, 120.4, 55.2, 49.5, 37.1, 35.9, 30.7. Anal. calc. for: C_24_H_21_N_3_O_2_ C, 75.18; H, 5.52; N, 10.96 %. Found: C, 74.89; H, 5.31; N, 11.20 %.

*2-(1-(1H-perimidin-2-yl)-2-phenylethyl)isoindoline-1,3-dione* *(5c)*

Brown solid, Yield 80 %; IR (KBr): ν_max_ 3300, 3059, 2920, 1713, 1636, 1468, 1385, 1242. ^1^H-NMR (DMSO-*d*_6_, ppm): δ 8.29 (s, 1H, N-H), 7.85-6.93 (m, 15H, H_aromatic_), 5.59 (m, 1H, CH-N), 3.49-3.44 (m, 2H, CH_2_).^ 13^C-NMR (DMSO-*d*_6_, ppm): 167.0, 135.3, 134.0, 130.9, 129.0, 128.9, 128.6, 128.5, 123.6, 123.5, 109.1, 108.7, 108.6, 51.5, 33.8. Anal. calc. for: C_27_H_19_N_3_O_2_ C, 77.68; H, 4.59; N, 10.07 %. Found: C, 77.42; H, 4.78; N, 10.31 %.

*2-(2-(4-hydroxyphenyl)-1-(1H-perimidin-2-yl)ethyl)isoindoline-1,3-dione* *(5d)*

Brown solid, Yield 70%; IR (KBr): ν_max_ 3334, 3192, 3057, 1711, 1595, 1470, 1383, 1288, 1177. ^1^H-NMR (DMSO-*d*_6_, ppm): δ 9.44 (s, 1H, O-H) 8.67 (s, 1H, N-H), 7.28-6.55 (m, 14H, H_aromatic_), 5.42-5.26 (m, 1H, CH-N), 4.07 (m, 2H, CH_2_).^ 13^C-NMR (DMSO-*d*_6_, ppm): 161.5, 146.1, 143.5, 139.4, 138.7, 136.0, 135.1, 127.3, 125.9, 125.5, 121.3, 120.5, 118.2, 117.3, 115.9, 110.0, 107.9, 107.8, 51.1, 31.5, 26.4. Anal. calc. for: C_27_H_19_N_3_O_3_ C, 74.81; H, 4.42; N, 9.69 %. Found: C, 75.04; H, 4.23; N, 9.84 %.

*2-(3-methyl-1-(1H-perimidin-2-yl)butyl)isoindoline-1,3-dione* *(5e)*

Black solid, Yield 70%; IR (KBr): ν_max_ 3239, 2951, 1707, 1578, 1449, 1368, 1275. ^1^H-NMR (DMSO-*d*_6_, ppm): δ 14.56 (s, 1H, S-H) 8.73 (s, 1H, CH=N), 7.68-7.45 (m, 5H, H_aromatic_),^ 13^C-NMR (DMSO-*d*_6_, ppm): 167.8, 154.3, 134.9, 134.7, 134.6, 131.5, 131.2, 131.0, 123.3, 123.2, 121.5, 56.0, 50.8, 37.1, 36.8. Anal. calc. for: C_24_H_21_N_3_O_2_ C, 75.18; H, 5.52; N, 10.96 %. Found: C, 74.99; H, 5.74; N, 10.88 %.

*α-Glucosidase inhibition assay*

In this study the inhibition study of both yeast and mammalian *α*-glucosidase were measured through the earlier reported bioassay method with minor changes (4). This test was carried out using the substrate *p*-nitrophenyl-*α*-D-glucopyranoside (pNPG), which is hydrolyzed by *α*-glucosidase to release the product *p*-nitrophenol, a color agent that can be monitored at 405 nm. For yeast *α-*glucosidase, mixture contained 20 µL of the enzyme (0.5 unit/mL), 120 µL of 0.1 M phosphate buffer (pH 6.9) and 10 µL of test sample at various concentrations. The mixed solution was incubated in 96-well plates at 37 °C for 15 min. After preincubation the enzymatic reaction was initiated by adding 20 µL of 5 mM pNPG solution in 0.1 M phosphate buffer (pH 6.9) and the reaction mixture was incubated for another 15 min at 37 °C. The reaction was terminated by adding 80 µL of 0.2 M sodium carbonate solution and then absorbance reading was recorded at 405 nm by microplate reader. The reaction system without tested samples was used as control and the system without *α*-glucosidase was used as blank for correcting the background absorbance. The inhibitory rate of sample on α-glucosidase was calculated by the following formula:

$\mathrm{\% Inhibition}=\left(\frac{\mathrm{control absorbance}-\mathrm{sample absorbance}}{\mathrm{control absorbance}}\right)\times 100$

The mammalian *α*-glucosidase were prepared from rat intestinal acetone powder (1 g), which was extracted by 25 mL of 0.1 M phosphate buffer (pH 6.9) containing 5 mM EDTA at ambient temperature. After sonication (10 min) and centrifugation (10000×g, 20 min) the obtained supernatant was dialyzed against 0.01 mM phosphate buffer (pH 6.9) for 24 h. The final supernatant was used for the assay. The mammalian *α*-glucosidase inhibition test was carried out in the same manner as that of the yeast *α*-glucosidase. 

## Result and Discussion

*Chemistry of synthesised compounds*

In a continuation of our previous investigations about benzimidazoles and pyrimidines synthesis, we have studied to prepare these hetrocycles with some protected amino acids which we obtained for the first time. 

In the first step, amino acids were chosen as starting material. Phthalic anhydride was reacted with amino acids to protect the amino group of them and converted to N-phthaloyl-L-amino acids (Scheme 1). Secondly, to synthesis the benzimidazole and pyrimidine derivatives, N-phthaloyl-L-amino acids were reacted with* O*-phenylendiamine or 1,8-naphtalendiamine respectively in POCl_3_. Synthetic pathway of the compounds is given in Schemes 2 and 3. 

The formulas of prepared compounds 3a-d and 5a-e were determined by elemental analyses and their structures were confirmed by IR, ^1^H-NMR and ^13^C-NMR spectral data. 

In the IR spectra N-H, C=O, C=N and C=C functions absorbed strongly in the expected regions: N-H at 3300-3400 cm^-1^, C=O and C=N at about 1550-1720 cm^−1^ and C=C at 1400 cm^-1^. IR spectra of N-phthaloyl-L-amino acids observed a broad absorption band at above 3000 cm^-1^ for O-H bond of COOH whereas the lack of this band in the spectra of the all products confirms complete condensation. Moreover a strong absorption band at about 1700-1800 cm^-l^ due to the stretching vibration C=O band of COOH group that obscured in benzimidazole and pyrimidine derivatives.

The ^1^H-NMR spectra showed aromatic protons at 6.00-8.00 ppm, as multiplet, in all of the compounds. In the compounds 3a-d, the N-H proton of benzimidazoles was observed at about 12.00 ppm as singlet. Whereas in the compounds 5a-e, the N-H proton of pyrimidines was appeared at about 8.00 ppm as singlet. The C-H proton between two aromatic rings was appeared at about 5.00-6.00 ppm in all products. All the other aliphatic protons were observed at the expected chemical shifts.

*α-Glucosidase inhibitory* *effect of synthetic compounds*

*α*-Glucosidase inhibitors are a group of antidiabetic drugs that are used for the control of diabetes mellitus type 2. The compounds decrease the absorption of carbohydrates from the digestive tract, thereby lowering the after-meal glucose level. In this study the inhibitory effect of new derivatives of benzimidazole and pyrimidine heterocycle compounds which synthesized with various aliphatic and aromatic amino acids were examined. Inhibitory effects of the synthesized compounds were screened using two kinds of *α*-glucosidase enzymes. Yeast *α*-glucosidase (type I), which has been frequently used as a primary model to investigate the inhibitory activity of potential inhibitors and rat intestinal *α*-glucosidase (type II), that serves as a better target to design and develop of antihyperglycemic agents (43). There are many reports that show very active yeast *α*-glucosidase inhibitors exhibit weak inhibitory effect on mammalian *α*-glucosidase (43,44). Indeed, the *α*-glucosidase activity of rat intestinal acetone powder nearly imitates the mammalian system, so may be a better model to identify and develop antihyperglycemic agents (45). Therefore in this study the inhibitory effect of synthetic compounds against rat intestinal *α*-glucosidase was also tested. The IC_50_ values of each compound for inhibition of yeast and rat intestinal *α*-glucosidase are shown in Table 2. IC_50_ values are the concentrations of synthetic compounds resulting in 50 percent inhibition of *α*-glucosidase enzyme activity. The values were determined by plotting a percent inhibition vs. concentration of these inhibitors. The results of this study indicate that among the benzimidazole derivatives, 4c and 4d compounds which synthesized from aromatic amino acids like phenylalanine and tyrosine respectively, possess significant inhibitory effect on the activity of both yeast and rat intestinal *α*-glucosidases. The best enzyme inhibitory effect is related to the 4d with the IC_50 _values of 9.1 and 36.7 µM for yeast and rat intestinal *α*-glucosidases, respectively. Also, 4c exhibited high inhibitory effect against yeast and rat intestinal with the IC_50 _values of 15.2 and 48.5 µM, respectively. The benzimidazole derivatives (4a and 4b) which contain aliphatic amino acids (glycine and isoleucine) showed almost negligible inhibition against both tested *α*-glucosidases. 

About the pyrimidine derivatives, 5c and 5d compounds with phenylalanine and tyrosine respectively, as the amino acid moiety were proven to be the most promising inhibitors of α-glucosidas. The IC_50 _values of 5c and 5d against yeast *α*-glucosidas were 11.9 and 8.3 µM, respectively, and these values against rat intestinal *α*-glucosidas were 35.9 and 21.8 µM, respectively. As it can be seen in Table 1, the other synthetic pyrimidine derivatives, with glycine, leucine and isoleucine as amino acid moiety (5a, 5b and 5e respectively) did not exert good inhibition activity on the tested *α*-glucosidases. With an attention to the inhibition activities of the synthetic compounds, it can be suggested that aromaticity play a significant role to improve the inhibitory properties of the compounds. In the both of benzimidazole and pyrimidine derivatives, the compounds with aromatic amino acids moieties (4c, 4d, 5c and 5d) exhibited significantly higher inhibitory action than acarbose against yeast and mammalian *α*-glucosidases. Indeed, it can be concluded that the aromatic domination of amino acid moiety on the heterocyclic scaffold may play important role in the inhibitory effect of these compounds. Also, with comparing the IC_50_ values of 4c and 4d as well as 5c and 5d against yeast and mammalian *α*-glucosidases, it can be suggested that hydrogen bonding ability is one of the effective parameters to ameliorate the inhibitory action of the synthetic compounds. 

**Table 2:** IC_50_ values of benzimidazole and pyrimidine compounds for inhibition of yeast and rat intestinal *α*-glucosidase.

**Compounds**	**IC**_50_ ** (µM)**	
	**Yeast**	**Rat**
**4a**	54.6±2.2	na
**4b**	na	na
**4c**	15.2±1.7	48.5±2.3
**4d**	9.1±1.2	36.7±2.9
**5a**	49.6±2.7	na
**5b**	na	na
**5c**	11.9±1.5	35.9±2.3
**5d**	8.3±0.9	21.8±1.7
**5e**	na	na
**Acarbose**	120.3±2.8	37.5±1.4

na: no activity

According to the mentioned explanations, the synthetic compounds with phenylalanine and tyrosine amino acids moieties (4c, 4d, 5c and 5d) with promising inhibitory properties on both yeast and rat intestinal *α*-glucosidases, may represent new class of hypoglycemic agents.

## Conclusion

We have designed and synthesized a series of novel 2-substituted benzimidazole and pyrimidine derivatives via the cyclization reaction of protected amino acids with *O*-phenylendiamine and 1,8-naphtalendiamine. All synthesized compounds were confirmed by FT-IR, ^1^H NMR and ^13^C-NMR and elemental analysis. Also the synthetic compounds were screened for their inhibitory effect on yeast and rat intestinal *α*-glucosidases and compounds 4c, 4d, 5c and 5d displayed significant ability on inhibition of the enzymes. Therefore, these compounds, open new avenues for the development of anti-hyperglycemic therapeutics agents to the treatment of type 2 diabetes.
